# A case series of cerebral venous thrombosis as the first manifestation of homocystinuria

**DOI:** 10.1177/23969873211059479

**Published:** 2021-11-12

**Authors:** Antonio Ochoa-Ferraro, Subadra Wanninayake, Charlotte Dawson, Adam Gerrard, Mary Anne Preece, Tarekegn Geberhiwot

**Affiliations:** 1 Department of Inherited Metabolic Disorders, 1732 Queen Elizabeth Hospital Birmingham, Birmingham, UK; 2 Department of Newborn Screening and Biochemical Genetics, 1729 BWC NHSFT, Birmingham, UK; 3 Institutes of Metabolism and Systems Research, 150183 University of Birmingham, Birmingham, UK

**Keywords:** Sagittal sinus thrombosis, pulmonary embolism, homocystinuria, cerebral venous thrombosis, lens dislocation, case series

## Abstract

**Background:**

Cerebral venous thrombosis (CVT) is an important cause of stroke particularly in younger patients and potentially fatal if diagnosis is delayed. The presentation of symptoms is highly variable and consequently the diagnosis and underlying cause is often delayed or overlooked. Homocystinuria, a rare autosomal recessive disorder is an identified risk factor for CVT.

**Purpose:**

A timely diagnosis and treatment of the underlying cause of CVT could result in improved outcome and prevent further events. This case series describes the clinical course of six adults presented with unprovoked CVT, in whom the diagnosis of underlying homocystinuria was delayed with adverse consequences. We aim to highlight the importance of recognising homocystinuria as an underlying cause of CVT and offer a practical approach to the diagnosis and management.

**Methods:**

This is a retrospective case series of a cohort of 30 consecutive patients seen in a UK tertiary referral centre.

**Result:**

Six out of 30 patients presented with CVT prior to homocystinuria diagnosis. The mean and range of age at the time of the first CVT episode was 22.6 (range 11–31) years. The mean ±SD age at diagnosis of homocystinuria as the underlying cause was 26 ± 4.2 years. The time between first CVT and diagnosis of homocystinuria ranged from 1.6 to 11 years resulting in a delay to introduction of effective treatment and, in some cases, a further large vessels thrombotic event.

**Conclusion:**

Physician awareness of homocystinuria as an underlying cause for an unprovoked CVT will facilitate timely introduction of effective treatment to prevent a further event.

## Introduction

Cerebral venous thrombosis (CVT) is a rare disorder, accounting for <1% of all strokes.^1^ Its estimated annual incidence in Western Europe is 1.32:100,000, but its worldwide incidence is variable.^2–4^ Thrombosis of the cerebral veins and sinuses, unlike arterial stroke, most often affects young adults and females.^2^ The symptoms and clinical course are highly variable and pose diagnostic challenges requiring imaging studies to confirm the diagnosis.^2,5^ Although there is a known multifactorial aetiology for CVT ranging from local to systemic risk factors,^5^ for 12.5% of adult CVT no underlying diagnosis is identified.^6^ However, hyperhomocysteinemia, due to acquired and genetic causes, is a recognised risk factor for CVT.^5–7^

**Figure 1.**
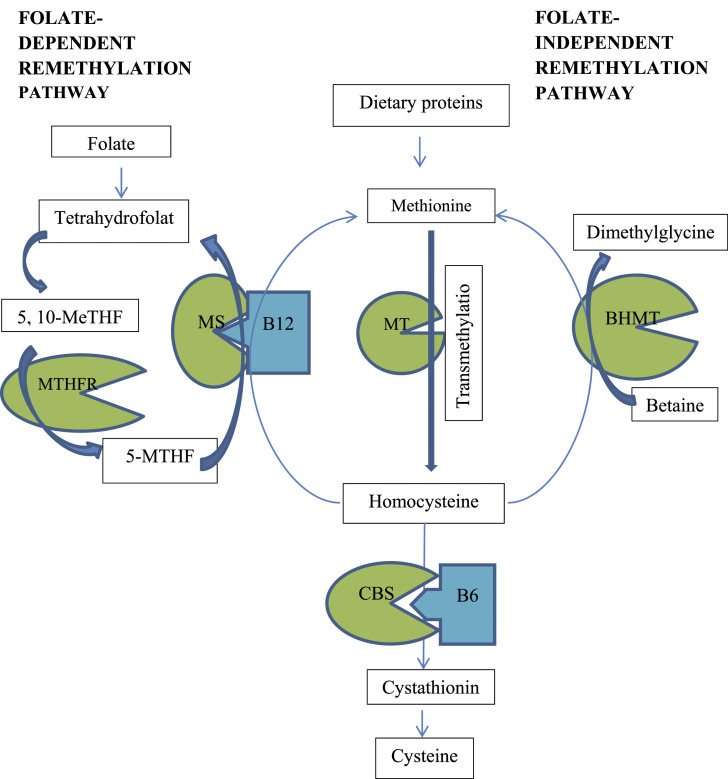
Metabolic pathway of homocysteine. Homocysteine is produced through transmethylation of methionine. It is metabolised into cysteine by vitamin B6 (B6)–dependent cystathionine β-synthase (CBS). Remethylation of homocysteine to methionine occurs in a folate dependent pathway (that is catalysed by vitamin B12 (B12) dependent methionine synthase (MS) with involving folate dependent methylenetetrahydrofolate reductase (MTHFR)) or a folate independent pathway (that is catalysed by betaine-homocysteine methyltransferase (BHMT). 5-MTHF indicates 5-Methylenetetrahydrofolate; 5,10-MeTHF, 5,10-Methylenetetrahydrofolate; MT, Methyltransferase.

Classical homocystinuria (HCU) (OMIM #236200) is a rare autosomal recessive metabolic disorder caused by defective activity of cystathionine beta-synthase (CBS), a pyridoxine (vitamin B6)-dependent enzyme responsible for converting homocysteine (Hcy) to cysteine via cystathionine.^8^ Remethylation of Hcy to methionine (Met) by either the cobalamin (vitamin B12)-dependent enzyme, methionine synthase, using 5-methyltetrahydrofolate as a methyl donor or betaine-homocysteine methyltransferase is an alternative pathway of Hcy metabolism (Figure 1).^8^

Classical HCU can manifest with a wide spectrum of age and severity, from multi-systemic early childhood onset to asymptomatic into adulthood. Patients might present in the first or second decade of life with ophthalmological (myopia, ectopia lentis), neurological (neurodevelopmental delay), skeletal (Marfanoid habitus) and haematological (thromboembolic) complications (Table 1).^8,9^ Worldwide prevalence is estimated to be at least 1 in 200,000–335,000, although it varies by ethnicity (1 in 65,000 in Ireland, 1 in 17,800 in Germany and 1 in 1800 in Qatar).^8,10^ The actual prevalence of HCU may be underestimated with many individuals diagnosed late in life following cardiovascular events (including strokes, pulmonary embolism (PE) and myocardial infarction) or ocular disorders.

**Table 1.** Clinical features, diagnosis, managements and complication.^8,9^

Clinical presentation		
	Common	Less common
Eye (≈85% of cases)	Lens dislocation	Glaucoma
	Myopia	
Skeleton (≈37% of cases)	Excessive height and length of the limbs (dolichostenomelia and arachnodactyly)	Bone deformities: Pectus excavatum, carinatum, genu valgum, scoliosis, long and slender fingers
	Osteoporosis	
Central nervous system (≈50% of cases)	Developmental delay/intellectual disability	Seizures, psychiatric and behavioural problems and extrapyramidal signs
Vascular system	Thromboembolism	
Diagnosis		
Plasma total homocysteine (μmol/L): High − typically >100	Methionine: High/ high normal	Cysteine: Low/ low-normal
Confirmatory testing: Measurement of cystathionine synthase activity in fibroblasts or plasma and/or by mutation analysis of the CBS gene		
Biochemical treatment targets		
Target plasma tHcy concentration less than 100 μmol/L		
Management		
Medication		
Pyridoxine-responsive: High dose pyridoxine	Partially pyridoxine-responsive: High dose pyridoxine, betaine	Pyridoxine-unresponsive: Betaine +/− low protein diet
Diet		
Low protein diet with Hcy-free amino acid supplementation if Hcy level not at target on medication alone (determined on an individual basis) or plasma methionine exceeding 1000 µmol/L to prevent use of higher dose of betaine		
Monitoring		
Biochemical	Plasma tHcy and methionine; amino acid profile for patients on a low protein diet (to assess adequacy of dietary protein); folate and vitamin B12 if non-adherence suspected	
Clinical	As clinically indicated, to include regular ophthalmology assessments, bone densitometry, cardiovascular and psychology	

CBS, Cystathionine beta-synthase; HCy, Homocysteine; tHcy, total homocysteine.

Elevated plasma total Hcy usually >100 µmol/L without co-existing nutritional deficiency supports the diagnosis of HCU. Accompanying high or borderline high methionine enhances confirmation of a diagnosis of CBS deficiency^8,11^ over other enzyme defects causing hyperhomocysteinemia. The aim of treatment is to reduce the plasma Hcy level to a desired range, making it one of the few potentially treatable inherited metabolic disorders (Table 1). In approximately 50% of patients, elevated Hcy responds either partially or fully to pyridoxine. Additional measures for lowering Hcy include betaine which acts as an alternative methyl group donor for remethylation (Figure 1). In a proportion of patients with severe childhood onset forms of HCU dietary protein restriction is also necessary.

Although we and others have previously published case reports of ‘cerebral venous thrombosis as the first presentation of classical HCU’^12–15^, there is no published evidence to highlight the magnitude of the problem. To our knowledge, this is the first case series study to present CVT as a primary manifestation of CBS deficiency.

## Method

This retrospective, observational study was conducted at the University Hospitals Birmingham NHS FT regional service for adult inherited metabolic disorders. We undertook a patient record search of our database to identify those patients with HCU caused by CBS deficiency who had CVT prior to their diagnosis of HCU. Six patients were identified of a total of 30 consecutive patients with HCU. Patients without CVT or those with a known diagnosis of HCU prior to CVT (two patients) were excluded. Patients have consented for their anonymised data to be published.

## Results

Patients’ characteristics and clinical course are shown in Table 2. The mean ±SD age at the time of the first CVT episode was 22.6 ± 7.5 years. The mean ± SD age at diagnosis of HCU as the underlying cause was 26 ± 4.2 years. The period from the first CVT episode until diagnosis of HCU as a cause varied from 1 month to 11 years. Four patients were female. Three patients had two CVT episodes each (1, 2 and 9 years gap between two episodes) and the rest a single event. There were two patients with other thrombotic events, namely PE and deep vein thrombosis. All patients with CVT had headache and seizures as their presenting feature. Marfanoid habitus was recorded in two patients. Bilateral lens dislocation in one patient was known prior to CVT and in three other patients, lens dislocation came to light as part of their HCU ophthalmology assessments. Intellectual disability was present in two patients. The mean ± SD of follow up after diagnosis is 8 ± 5.62 years (Range 1–18 years).

**Table 2.** Patient characteristics, clinical presentation and outcome.

Patient	1, M		2, F		3, F	4, F	5, M		6, F
Current age (years)	29		40		27	44	44		19
Presentation during first admission	Severe headaches and repeated episodes of seizure		Severe headache, dysarthria and right hemi-paresis		Severe headache and seizure	Headaches, sustained grand mal epileptic fit	Severe headaches, blurred vision and episodes of seizures		Headache, seizure and papilloedema
Age in year at clinical presentation of CVT (years)	25.4	27.5	31	32	16	19	26	35	11
Delay in underling cause diagnosis (years)	3.6		1.6		6	7	11		0.1
Bilateral lens dislocation	YES		YES		YES	YES	NO		YES
Learning difficulties	NO		NO		Mild	NO	NO		Mild
Other thrombotic event	NO		PE (31yo)		DVT (27yo)	NO	NO		NO
Marfanoid body habitus	NO		NO		NO	YES	NO		YES
Pregnancy/Oral contraceptive	Not applicable		YES – Oral contraceptive		NO	NO	Not applicable		NO
Other identified risk factors for CVT	NO		NO		NO	NO	NO		NO
Total homocysteine - pre-treatment (μmol/L)	>350		>350		178	200	331		>350
Methionine pre-treatment (μmol/L)	61		31		Not available	88	80		153
Total homocysteine - post treatment (μmol/L)	42		10		52	70	57		98
Treatment	Pyridoxine + Folate		Pyridoxine + Betaine + folate + Hydroxocobalamin		Pyridoxine + Betaine + folate + Hydroxocobalamin	Pyridoxine + Betaine + folate + Hydroxocobalamin	Pyridoxine + Betaine + folate + Hydroxocobalamin		Betaine + low-methionine-diet

CVT, cerebral venous thrombosis; DVT, deep vein thrombosis; PE, pulmonary embolism.

### Case Descriptions

Case 1 A 25 year old male was admitted to hospital with severe headache and seizure, no history of trauma; magnetic resonance imaging (MRI) indicated haemorrhagic stroke. 2 years later, he had another generalised tonic clonic seizure and further brain MRI/MRA showed central venous thrombosis. He was treated with oral anticoagulant (warfarin) for approximately 10 months after which the risk of stroke was perceived to have been mitigated, and maintained on Levetiracetam 500 mg BD to prevent seizure recurrence. At age 29, bilateral lens subluxation and rapidly progressive myopia from −1.2 to −8.5 dioptre alerted his ophthalmologist to suspect HCU. Total Hcy on referral was >350 µmol/L.

Case 2 A 31 year old female presented with severe headache, dysarthria and right hemi-paresis followed by right-sided focal myoclonic seizure. She was treated for suspected viral encephalitis. After 2 months, she presented with chest pain and breathlessness due to radiologically proven PE. In the following year, she was re-admitted with increasing frequency of headaches and repeated magnetic resonance imaging (MRI) and MRA head scan was consistent with sagittal venous sinus thrombosis. Although initial thrombophilia screening was negative, subsequent investigations for an underlying cause revealed an elevated total Hcy >350 µmol/L with normal plasma folate and methylmalonic acid (MMA) concentrations. Ophthalmological screening confirmed bilateral lens subluxation. An underlying diagnosis of HCU secondary to CBS deficiency was confirmed. Since treatment for HCU commenced 10 years ago she has had no further thrombotic event.

Case 3 A 16 year old female was found to have radiologically confirmed cerebral sagittal sinus thrombosis following presentation with severe headache and seizure. She was anticoagulated. 6 years later, she presented with visual disturbance and lens subluxation leading to the diagnosis of classical HCU. She has had two miscarriages; the first 2 years prior to diagnosis of HCU and a subsequent one 2 years later when plasma Hcy control was poor due to non-adherence with treatment. After achieving satisfactory Hcy level in the same year, she had a successful pregnancy with uneventful postpartum course. During the period she was non-adherent to her treatment with elevated plasma Hcy she developed a venous thrombosis in the left greater saphenous vein.

Case 4 A 20 year old female was admitted with progressive headaches followed by sustained grand mal epileptic seizure and treated for viral encephalitis. She was born with bilateral lens dislocation and noted to have a Marfanoid body habitus and diagnosed as Marfan’s syndrome at the age of 12. However, at the aged of 27 years, not having a causative mutation for Marfan’s Syndrome in the Fibrillin-1 gene, skin biopsy was performed and confirmed CBS deficiency consistent with classical HCU. In addition, recent brain MRI scan revealed hyper-intense signal change along the anterior aspect of the superior sagittal sinus consistent with old sagittal sinus thrombosis.

Case 5 A 26 years old male presented with progressive headaches followed by grand mal epileptic seizure, no evidence of lens dislocation, learning difficulties or Marfanoid body habitus. Imaging of the brain confirmed thrombotic changes in the region of the torcular and along the superior sagittal sinus, straight sinus and vein of Galan. He was anticoagulated for about 4 years. At 35 he presented with severe progressive headaches and grand mal epileptic seizure. CVT was confirmed and anticoagulation was restarted. Subsequent investigations revealed high Hcy and Met with low cystine. Hcy normalised after successful treatment and anticoagulation was discontinued.

Case 6 An 11 year old girl was admitted with headache, seizure and papilloedema. CVT was confirmed on brain imaging showing intracranial haemorrhage in the right frontal lobe in addition to the sagittal venous sinus thrombosis. Investigating for a secondary cause identified high Hcy level of >350 μmol/L, consistent with the diagnosis of HCU. Following her HCU treatment she has had no similar incident in the last 8 years.

## Discussion

Unprovoked CVT remains a diagnostic challenge and a potentially lethal or disabling clinical event, but improved diagnosis and treatment of the underlying cause could alter the clinical course and prevent further life threatening thrombotic events. In this case series study of six cases of CVT caused by untreated HCU, we illustrate the importance of recognising a treatable condition and for effective prevention of further episodes. In fact, a significant proportion of patients with HCU (eight in total, two after diagnosis of HCU) in our study sample had at least one episode of CVT. The mean age of CVT event in general population has been reported at 39 years.^6^ However, the mean age of CVT in untreated HCU in our study is 22.6 (±7.5) indicating a preponderance to even younger ages.

The wide spectrum of clinical presentations creates difficulties for the diagnosis of CVT. As a result, a high index of suspicion, greater clinical awareness and more sensitive neuroimaging techniques are essential for the differentiation of affected individuals from other neurological diseases.^3^ Similarly, in three out of six patients, CVT was misdiagnosed at their initial presentation resulting in missed opportunity to offer appropriate treatment and prevent further thrombotic event. In this study, 100% had severe headache and seizures and all are unprovoked with no identifiable risk factor. Four of our patients had a history of either visual disturbance or lens problem and careful ophthalmologic history could have shed light on the underlying cause of CVT. Like CVT, HCU in adults poses a diagnostic challenge as it predominately manifests with either large vessel thrombotic events or lens dislocation without the classical textbook description of Marfanoid body habitus or intellectual disability. Clinical awareness facilitates the correct diagnosis of HCU when presented with CVT. In case 6, a childhood-onset CVT, Marfanoid body habitus and intellectual disability facilitated the diagnosis of HCU without delay. However, in all adult patients there was a 1.6–11 (mean ± SD 5.9 ± 4.2) years’ delay between initial presentation and diagnosis of HCU. Interestingly, detection of lens dislocation, the ocular hallmark of the disease,^8^ facilitated the HCU diagnosis in case 2, while in the remainder, lens dislocation was identified only after the biochemical diagnosis.

Systemic diseases such as inflammatory diseases, malignancies and local factors including surgery, infections, trauma and tumours are common causes for CVT.^3,6^ In addition, inherited and acquired thrombophilias are acknowledged to increase the risk of venous thromboembolism (VTE).^5^ It is important to acknowledge hyperhomocysteinemia is a relatively weak prothrombotic factor. However, this changes dramatically with the very high homocysteine levels caused by CBS deficiency. The mechanisms of thrombus formation in hyperhomocysteinemia are poorly understood but proposed mechanisms include a homocysteine-mediated enhanced platelet activation; decreased expression of the anticoagulant protein thrombomodulin, which is essential for activation of anticoagulant protein C, and a relative lack of endothelium-derived nitric oxide (NO) leading to endothelial dysfunction.^16^

**Figure 2.**
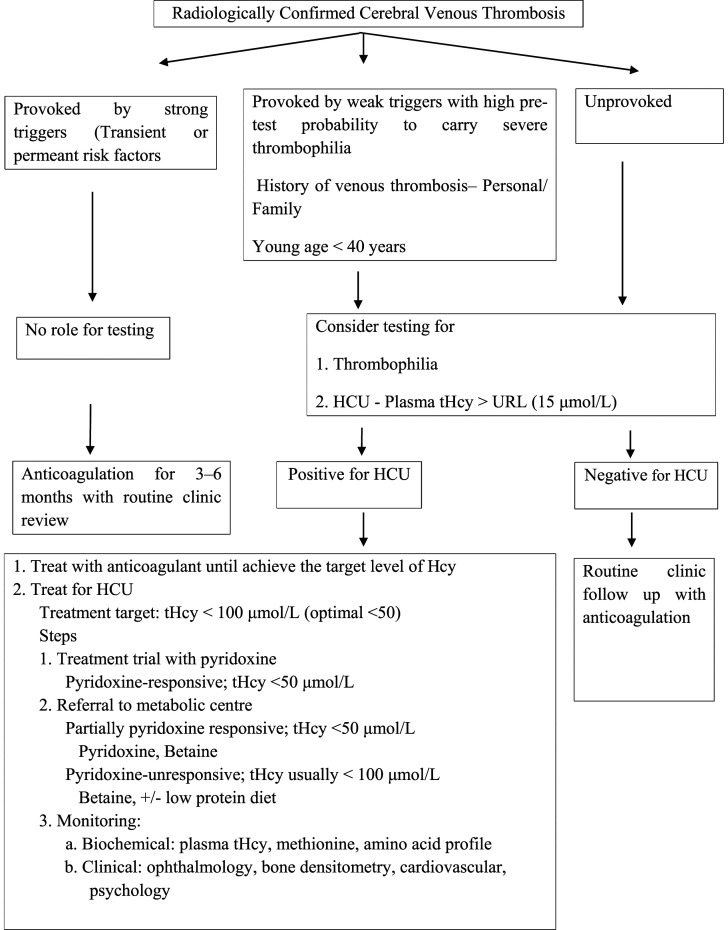
Proposed algorithm for selecting patients with First CVT to test for HCU and management. CVT, cerebral venous thrombosis; HCU, classical homocystinuria; tHcy, total homocysteine; URL, upper reference limit.

Both the American Heart Association and American Stroke Association and the European Stroke Organisation guidelines recommend thrombophilia screening at least in certain circumstances after confirming CVT by imaging study to prevent recurrent venous thrombotic events.^2,17^. On the other hand, although hyperhomocysteinemia is a well-known risk factor for CVT,^18–21^ there is a gap in thrombosis management guidelines not including Hcy measurements. We think measurement of plasma Hcy along with thrombophilia screening will enhance the diagnostic pick up of an underlying cause of unprovoked CVT or any large vessels thrombosis and propose modification of the current guideline as per Figure 2. In brief, clinical characteristics suggestive of HCU in patients with VTE; VTE in conjunction with weak provoking factors at a young age, often considered to be less than 40 years of age; VTE in an unusual site such as the central nervous system; recurrent VTE events, and eye glass requirement at young age or history of lens dislocation.

In addition, although there is a new-born screening programme for detecting CBS deficiency in many countries, the sensitivity of methionine based method (employed in many counties) for detecting new-born with CBS deficiency is questionable. In fact, 20–50% of pyridoxine non-responsive cases may be missed and detection of pyridoxine responsive CBS is seldom in existing programmes.^8,15^ It is important that in this study, five out of six cases are at least partially pyridoxine responsive.

Testing for HCU is straightforward by measuring total homocysteine (tHcy) level in plasma. In CBS deficiency, plasma tHcy at diagnosis vastly exceeds by 10–20-fold the upper limit of the reference range (generally 10–15 μmol/L, but this varies with age and method of analysis). Although CBS deficiency should be confirmed by measurement of cystathionine synthase activity in fibroblasts or plasma and/or by mutation analysis of the CBS gene, neither technique can be relied on to demonstrate abnormalities in all cases.^8^ In all of our patients presented here, the Hcy concentration was greater than 178 µmol/L at the time of diagnosis, therefore exceeding the consensus diagnostic threshold for CBS deficiency.^8,22^ Therefore, the diagnosis of HCU would have come to light had tHcy been measured at the time of first presentation.

The treatment goal for HCU is to lower the plasma tHcy concentration to a level of less than 100 µmol/L at which further complications of thromboembolism can be prevented. At the time of diagnosis, responsiveness to pyridoxine should be assessed. There are various protocols for doing this.^8^ Patients may be either fully or partially pyridoxine responsive or non-responsive. Hence pyridoxine treatment alone may be sufficient to meet the therapeutic target or patients may need betaine in conjunction with pyridoxine if partially responsive or alone if non-responsive. It has been demonstrated that reduction of Hcy concentration to below the therapeutic target limits the severity of complications and reduces the risk of a further vascular event.^23^ In our study, 50% of patients had two episodes of large vessels VTE within 9 years during the undiagnosed period while no patients on treatment had a subsequent vascular complication during follow up (ranging from 1 to 18 years) except case 3 who had persistently elevated Hcy levels caused by non-adherence to treatment.

In conclusion, although HCU is very rare condition it is an important treatable and preventable cause of CVT or PE. Thus, testing for HCU is simple and effective for a young (40 years) patient presenting with unprovoked CVT and/or PE.

## Non-standard Abbreviations and Acronyms

CVT: Cerebral venous thrombosis
HCU: Classical homocystinuria
CBS: Cystathionine beta-synthase
Hcy: Homocysteine
Met: Methionine
VTE: Venous thromboembolism
tHcy: total homocysteine
PE: pulmonary embolism
